# From 2D to 3D Cancer Cell Models—The Enigmas of Drug Delivery Research

**DOI:** 10.3390/nano10112236

**Published:** 2020-11-11

**Authors:** Indra Van Zundert, Beatrice Fortuni, Susana Rocha

**Affiliations:** Molecular Imaging and Photonics, Chemistry Department, KU Leuven, Celestijnenlaan 200F, 3001 Heverlee, Belgium; indra.vanzundert@kuleuven.be

**Keywords:** nanoparticles, drug delivery systems, spheroids, 3D culture systems, 3D cell models

## Abstract

Over the past decades, research has made impressive breakthroughs towards drug delivery systems, resulting in a wide range of multifunctional engineered nanoparticles with biomedical applications such as cancer therapy. Despite these significant advances, well-designed nanoparticles rarely reach the clinical stage. Promising results obtained in standard 2D cell culture systems often turn into disappointing outcomes in in vivo models. Although the overall majority of in vitro nanoparticle research is still performed on 2D monolayer cultures, more and more researchers started acknowledging the importance of using 3D cell culture systems, as better models for mimicking the in vivo tumor physiology. In this review, we provide a comprehensive overview of the 3D cancer cell models currently available. We highlight their potential as a platform for drug delivery studies and pinpoint the challenges associated with their use. We discuss in which way each 3D model mimics the in vivo tumor physiology, how they can or have been used in nanomedicine research and to what extent the results obtained so far affect the progress of nanomedicine development. It is of note that the global scientific output associated with 3D models is limited, showing that the use of these systems in nanomedicine investigation is still highly challenging.

## 1. Introduction

Despite the significant advances in cancer therapy over the past decades, cancer remains the one of the major causes of death worldwide [1]. Surgery, chemotherapy and radiation therapy, the most commonly used cancer treatments, often fall short due to the incomplete removal of the cancerous tissue, the non-specificity of the therapy or development of multidrug resistance, among other causes [2]. Drug nanocarriers have the potential to address these limitations. The increasing interest in nanomedicines has led to tremendous progress in this research field. Nowadays, there is an immense offer of nanocarriers of different materials, sizes, shapes and various surface modifications, for increased stability and active targeting of the cancer cells [3,4,5,6]. However, only a handful of cancer nanomedicines have reached the clinical stage. Among them, the most known examples are Doxil^®^ and Abraxane^®^, for the delivery of doxorubicin and paclitaxel, respectively [7,8].

In spite of this progress, promising results obtained in standard 2D cell culture systems often turn into disappointing outcomes in in vivo models. In a research laboratory, the efficacy and cytotoxicity of nanomedicines are almost exclusively studied on two dimensional (2D) monolayer culture systems, as this is the most straightforward and low-cost approach. However, the promising results obtained in 2D monolayer cultures often cannot be reproduced in vivo, using animal models [9]. This discrepancy can be attributed to the enormous gap between the 2D culture systems commonly used and the actual physiological situation in mammalian bodies. Two-dimensional monolayer cultures are far too simplistic to mimic the complex microenvironment of a tumor. More specifically, the presence of a three-dimensional (3D) network of different cell types, surrounded by extracellular matrix (ECM), cannot be recapitulated by 2D monolayer cultures. Nevertheless, both the 3D cell arrangement and the ECM are known to play a crucial role in the diffusion and cellular uptake of nanoparticles in vivo [9,10]. 

While 2D culture systems remain the most popular screening method used for drug development, researchers have started exploiting more complex 3D model systems to bridge the gap between the in vitro models and in vivo physiology of the human body. Three-dimensional cell models can have different levels of complexity, from single cells in 3D scaffolds towards multicellular miniature tumors, derived from cancer patients (tumoroids) or even organ-on-chip systems (for an extensive summary on 3D cell culture systems, see references [11,12,13]). Despite the increased popularity of such complex 3D models, their application in the development and evaluation of drug nanocarriers remains limited and the majority of nanomedicine development is still performed on 2D monolayer cell culture systems. Recently, the groups of Mura and Stenzel have independently published two review papers on the use of multicellular tumor spheroids as a tool for the investigation of nanomedicines [14,15]; and Mapanao and Voliani provided an overview 3D tumor models available and discussed how these models contribute to the advance of nanotheranostics [16]. In this review, we will provide an overview of the different 3D cancer cell models currently available and discuss how they have been used in the field of nanomedicine. We will pinpoint the advantages and disadvantages of each model, explain how each model relates to the in vivo physiology of the tumor tissue and highlight the potential of each model to evaluate specific aspects of nanocarrier design. In addition, we will briefly discuss the existing characterization methods and (fluorescence) assays currently used. We aim to provide a guideline for researchers working in nanoparticles that wish to start using 3D cancer models for the development of nanocarrier-based cancer treatments. 

## 2. From 2D to 3D Cancer Models

Currently, there is a wide range of 3D cell models available for the design and evaluation of drug nanocarriers. While none of these models can fully mimic the complex tumor micro-environment, each one can be used to study specific aspects of the behavior of nanoparticles in vivo. In this review, we divided the different 3D cell models into three categories, namely scaffold-free, scaffold-embedded and microfluidic(s)-based models. 

### 2.1. Scaffold-Free 3D Model

**Figure 1:**
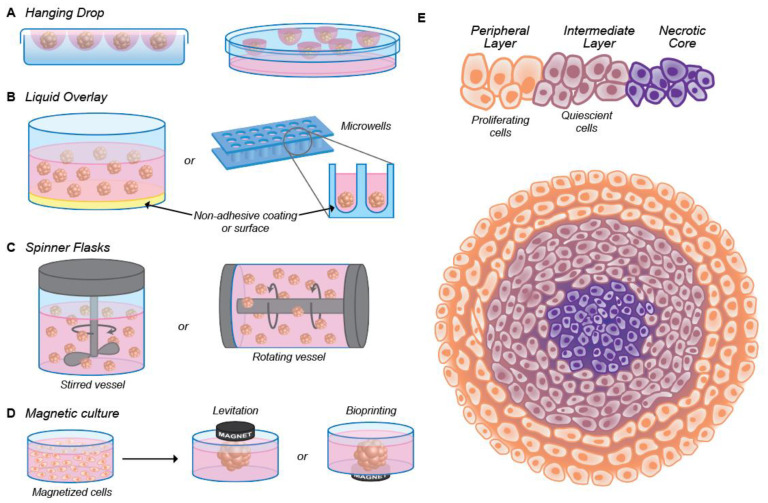
(**A**–**D**) Illustrative scheme of the methods for the preparation of multicellular tumor spheroid (MCTS):: the hanging drop method, where the cells come together at the bottom of a hanging drop (**A**), the liquid overlay method, where the surface of plates or (conical) wells is coated with a non-adhesive material (**B**), spinner flasks (stirred or rotating vessel), where the cells are kept in suspension (**C**) and magnetic culture, where, after the addition of magnetic particles, the cells are brought together through magnetic forces (**D**). (**E**) Schematic representation of the spheroid physiology depicting the distribution of proliferating (orange, outside layer), quiescent (violet, middle layer) and necrotic cells (purple, at the core). The cellular density is lower in the outside layer.

One of the most popular 3D cell models currently used is the multicellular tumor spheroid (MCTS) model [14]. Spheroids can be formed either via single cells that proliferate into cell aggregates or via pre-aggregated cell clusters that further proliferate. During cell proliferation, intercellular communication is established and the cells create their microenvironment [17,18,19]. The most commonly used methods to prepare spheroids are illustrated in Figure 1. These include:(i) the liquid-overlay technique (using coated surfaces or conical/U-shaped bottom wells), where the cells sink to the bottom of the conical well to aggregate and in turn form a spheroid [20]; (ii) the hanging-drop method, in which the cells come together at the bottom of a hanging drop [21]; (iii) spinner bioreactors, where cells are kept in suspension through the use of spinner flasks or rotating vessels [15]; and (iv) magnetic culture levitation, where the cells are brought together by magnetic forces after loading magnetic nanoparticles inside the cells [22] (Figure 1A–D). It is of note that in nanoparticle research, the use of magnetic cultures is not advisable as it requires magnetization of the cells with nanoparticles (a process that might affect other particle-cell interactions). In fact, in the majority of the reports where spheroids are used, the 3D cell aggregates are achieved using the liquid overlay technique, either by using conical/U-shaped bottom wells, agarose-coated surfaces or micro-molded non-adhesive hydrogels. A detailed overview of the preparation methods and comparison between the different preparation techniques can be found in the recent review of Velasco et al. [23] and references therein. 

Spheroid models have been proven to be more physiologically relevant than 2D monolayer cultures [14,24], recapitulating some key features of solid tumors. For instance, the growth kinetics of spheroids is similar to real tumors, where an outer layer of proliferating cells surrounds a layer of non-proliferating cells (quiescent cells), with the necrotic cells accumulating in the core of the structure (Figure 1E). In the outer layer of the spheroid, the cells are loosely attached to each other, whereas in the intermediate layer, cell packing and the ECM are denser [25,26]. As a consequence, an oxygen gradient arises due to impaired O_2_ diffusion, which is one of the causes of necrosis in the core region [25,27,28]. In addition, enhanced lactic acid fermentation leads to extracellular acidosis (lower pH). While in healthy tissue, the extracellular pH lies in the 7.3–7.4 range, the extracellular pH in the spheroid core generally presents a lower value (6.2–6.9) [29,30]. This is similar to what has been found in a solid tumor. The cellular heterogeneity of the tumor tissue can be partially mimicked by using co-cultures of cancer cells with fibroblasts or macrophages [31,32].

While scaffold-free spheroids are prepared in a culture medium, there is evidence that cells within the spheroids produce ECM proteins, such as fibronectin, laminin, collagen and glycosaminoglycans [33,34], that accumulate in the intercellular space. In addition to the 3D distribution of proliferative, senescent and necrotic cells, ECM deposition prompts the use of spheroids to investigate how the properties of nanocarriers influence their ability to penetrate and diffuse in solid tumors. Table 1 summarizes some of the publications where MCTSs were used to study the influence of different properties in the diffusion and penetration of nanoparticles. The most studied parameters, namely size, charge and surface functionalization, are discussed in more detail in the sections below. Some other properties of the nanoparticles might also affect their efficiency (e.g., stiffness, shape). Due to the limited number of studies, their influence will not be discussed in this review (a few relevant articles can be found in Table 1). For a more comprehensive overview of the different types of nanoparticles and their applications, see the recent review from Khan et al. [35].

#### 2.1.1. Nanoparticle Size 

**Figure 2:**
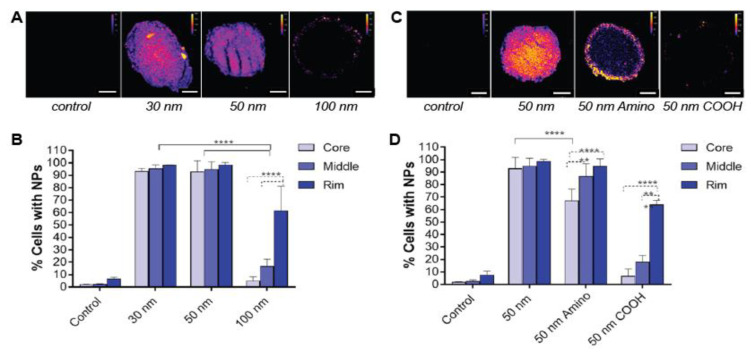
The effect of nanoparticle size (**A**,**B**) and charge (**C**,**D**) on penetration depth in spheroids. (**A**) Confocal images of 20 µm frozen sections of HCT116 spheroids after 24 h incubation with 30, 50 and 100 nm polystyrene nanoparticles. Scale bar: 100 µm. (**B**) The distribution of the different sized particles (30, 50 and 100 nm) across the spheroid, distinguishing the core, middle and rim. (**C**) Confocal images of 20 µm frozen sections of HCT116 spheroids after incubation with 50 nm polystyrene nanoparticles (unmodified, aminated and carboxylated polystyrene nanoparticles). Scale bar: 100 µm. (**D**) The distribution of the 50 nm particles with different surfaces (unmodified, aminated and carboxylated) across the spheroid, distinguishing the core, middle and rim (****, ** and * indicate *p* < 0.0001, *p* < 0.01 and *p* < 0.05, respectively). Adapted from Reference [37], with permission of American Chemical Society © 2020.

Over the last years, the spheroid model has been used to find the optimal size of nanocarriers for an enhanced penetration in solid tumors. Different studies have repeatedly shown that the penetration depth is inversely proportional to the size of the nano-carriers [36,38,60]. In the work of Hinger et al., the cellular uptake of 50 nm and 120 nm lipid nanocarriers was evaluated in CAL-33 spheroids (tongue squamous cell carcinoma cells). The lipid carriers used, designated by Lipidots, transported photosensitizers. These particles were designed for photodynamic therapy, where tumor cells are killed through the generation of cytotoxic reactive oxygen species by light irradiation. The 50 nm Lipidots penetrated deeper into the spheroids and presented a light-induced toxicity higher than the one induced by 120 nm Lipidots [36]. In a more recent study, performed by Tchoryk and co-workers, the penetration of 30, 50 and 100 nm fluorescently labeled polystyrene nanoparticles in HCT116 spheroids (human colorectal carcinoma cells) was evaluated using confocal microscopy and fluorescence-activated cell sorting (FACS) [37]. For 30 and 50 nm particles, after 24 h of incubation, 90% of the cells in the spheroids presented internalized particles. Differently, when 100 nm particles were used, only 22% of the cells contained particles after 24 h. Confocal microscopy revealed that 30 and 50 nm non-functionalized particles were evenly distributed throughout the whole spheroid (core, middle and rim) while 100 nm nanoparticles were found mainly at the periphery (rim) of the spheroid (Figure 2A,B). Together with the reports listed in Table 1, these results indicate that in terms of penetration depth “the smaller, the better.” This conclusion, drawn from studies using MCTS models, matches the results obtained using in vivo models. For instance, Tang et al. found that drug- conjugated PEGylated silica nanoparticles of 50 nm penetrate deeper into lymphoma (EL4 tumors collected from mice) than their 200 nm counterparts [61]. In another study, Huang and co-workers investigated the relationship between the size of drug-coated gold nanoparticles and particle penetration in vivo and in MCF-7 spheroid models (human breast adenocarcinoma cells). They reported that ultrasmall particles (diameter smaller than 10 nm) were able to penetrate deeply into tumor spheroids and showed accumulation levels in the tumor tissue higher than 15 nm particles [40]. When using ultrasmall nanoparticles in vivo, however, the efficient renal clearance of particles with a diameter smaller than 8 nm causes a drastically lower half-life in the body after injection [62,63]. In addition, it has been shown that nanoparticles smaller than 50 nm interact with hepatocytes [64]. 

**Figure 3:**
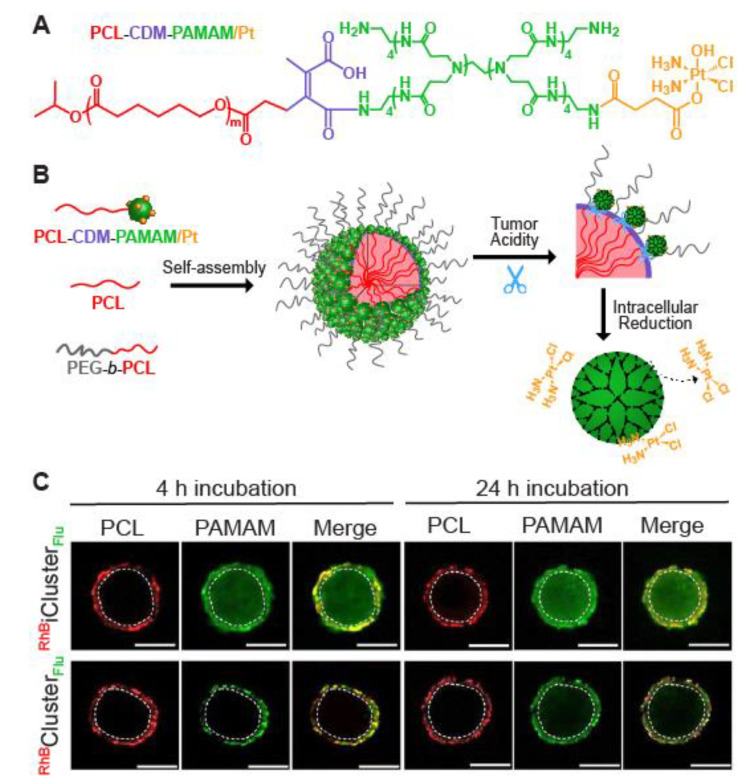
The design and penetration depth of clustered particles, with (iCluster) or without (control, Cluster) a degradable bond (2-propionic-3-methylmaleic anhydride, CDM). (**A**) Chemical structure of platinum (Pt) prodrug-conjugated poly (amidoamine)-graft-polycaprolactone (PCL-CDM-PAMAM/Pt). (**B**) Self-assembly and structural change of iCluster/Pt in response to tumor acidity and intracellular reductive environment. (**C**) In vitro penetration of ^RhB^iCluster_Flu_ and ^RhB^Cluster _Flu_ (cluster particles were dual-labeled with two dyes) in MCSs at pH 6.8 after a 4 h or 24 h incubation. The area marked with white circles was considered the inside area (Scale bar, 200 μm). RhB: Rhodamine B, Flu: Fluorescein. Adapted from Reference [43], with permission from PNAS © 2020.

According to the state-of-the-art of drug delivery systems, the targeting efficiency and accumulation in the tumor tissue are enhanced when the size of the drug nanocarriers is constricted to 50–60 nm in diameter. However, research using MCTSs indicates that nanoparticles with this dimension are often retained in the peripheral layers of the spheroid (penetration also depends on the surface functionalization). Different strategies have been devised to develop multistage (or size-switching) nanosystems, that initially have a size suitable for long blood circulation (50–200 nm) but upon a specific trigger, release smaller nanoparticles in a progressive manner [65]. Size-switching nanoparticles have been developed where the release is induced by near-infrared light [55], enzyme-mediated degradation [47,48] or low oxygen concentration [56]. However, the most commonly used trigger is the lower pH value of the extracellular environment, present in solid tumors and mimicked by the spheroid model. A good example of the latter approach are the stimuli-responsive nanoparticles developed by Li et al. [43]. These polymeric clustered nanoparticles (iCluster) had an initial size of ~100 nm, for longer blood circulation time and high accumulation at the tumor sites. The higher extracellular acidity present in the spheroid triggered the discharge of poly (amidoamine) dendrimers (diameter ∼5 nm), conjugated with a platinum prodrug. To evaluate the distribution in BxPC-3 spheroids (pancreatic cancer cells) with confocal microscopy, the polymeric component of the hydrophobic core was labeled with rhodamine B while the dendrimers contained fluorescein. While the bigger particles (∼100 nm) were retained at the periphery of the spheroids, the pH-mediated release of the dendrimers facilitated their penetration into the spheroid and subsequent cellular internalization of the therapeutic drug (Figure 3). 

#### 2.1.2. Surface Charge

In addition to their size, surface properties also influence the distribution of nanoparticles in the MCTS model. Several research groups have investigated the effect of the surface charge in nanoparticle uptake. Jin et al. reported that negatively charged gold nanorods (55 nm × 15 nm) could penetrate MCF-7 spheroids, whereas their positively charged counterparts accumulated only at the outer region. The more homogeneous distribution of the negatively charged nanorods in the spheroid maximized their photothermal cytotoxicity [42]. In a later study, Solomon et al., reported a similar behavior for two types of liposomes, one with a positive and one with a negative surface charge [60]. They observed an accumulation of the cationic liposomes in outside layer of the LLC spheroids (mouse Lewis lung carcinoma cells), attributed to the high cell binding ability, but they obtained a higher penetration depth for anionic liposomes. In the previously discussed report of Tchoryk and coworkers [37], they examined the effect of 50 nm positively charged (aminated), negatively charged (carboxylated) and unmodified polystyrene nanoparticles on HCT116 spheroid penetration (Figure 2C,D). They found pronounced differences in their uptake profiles. As such, the penetration of unmodified nanoparticles was found to be the fastest and the deepest towards the core of the spheroid (Figure 2), being internalized by over 90% of the spheroid cells after 24 h (determined by FACS). After 24 h, the aminated nanoparticles were also internalized by ~80% of the cells, however, the nanoparticles were mainly located at the periphery and penetration towards the core was limited, similar to what has been established in the earlier reports of Jin [42] and Solomon [60]. Differently, the carboxylated nanoparticles only reached a maximum of 22% of the spheroid cells at the periphery of the spheroid, which indicates no penetration of the negatively charged nanoparticles in the spheroid. In a recent work of Bugno and coworkers, positively (aminated) charged dendrimers were accumulated in the spheroid (KB, human epithelial carcinoma cells), penetrating towards the core while neutral and negatively (carboxylated) charged dendrimers remain on the peripheral layers [38]. 

Most of the results obtained so far suggest that nanoparticles with a positive charge bind strongly to the cell membrane and therefore stay at the periphery of the spheroid. There are, however, some contradictory results. Different research groups observed negligible penetration of carboxylated particles (negative charge). This contradiction indicates that in addition to the surface charge, the specific functional group added might also play an important role in the penetration and diffusion of the nanoparticles in multicellular structures. 

#### 2.1.3. Surface Functionalization 

Coating nanoparticles with polyethylene glycol (PEG) (or PEGylation) is a common strategy to increase blood circulation times, decrease nanoparticle aggregation and minimize opsonization [66]. PEGylation is the most popular stealth-shielding technology but there are others [67,68,69]. Of note, PEG can further be used as a scaffold for modification with ligands or other functional groups. An approach to increase the cell specificity and uptake of drug delivery systems is to functionalize their surface with specific ligands. Molecules that recognize and bind to membrane receptors (ligands) are often added to the surface of nanocarriers to specifically target cancer cells. For instance, nanoparticles coated with hyaluronic acid interact with CD44 receptor molecules, overexpressed in several cancer cell types [70]. Alternatively, the nanoparticle’ surface can be grafted with folate groups, which bind to the folate receptor. This receptor is highly abundant in various cancers such as ovary, uterus, endometrium and so forth. [71]. It is well known that functionalization with specific ligands, such as hyaluronic acid and folate, increases the cellular uptake of the nanocarriers in 2D cell culture systems. The effects of folic acid (FA) functionalization on the penetration of NPs has been evaluated using HeLa spheroids [54]. Nanoparticles coated with FA achieved considerable deeper penetration in comparison to non-targeted particles. Importantly, the amount of folate groups on the surface was tuned to yield particles with a zeta potential ±10 mV.

Another functionalization that has achieved promising results in particle penetration in the spheroid models is the addition of iRGD, a 9-amino acid cyclic peptide (sequence: CRGDKGPDC). The iRGD peptide binds to integrins on tumor endothelium, thereby facilitating tumor tissue penetration in vivo [72,73,74]. It has been repeatedly shown that nanoparticles functionalized with iRGD show higher accumulation and penetration into MCTS [49,50,51,56]. In addition, nanoparticles decorated with cell-penetrating peptides (CPPs) also present higher penetration. CPPS are short peptide sequences, mostly cationic, that promote the crossing of (macro-) molecules over the plasma membrane [75,76,77]. Recently, Van Oppen and coworkers functionalized elastin-like polypeptide nanoparticles (60 nm) with octa-arginine peptides (R8) and investigated the influence of the peptide density (0%, 10% and 25%) on the penetration depth and cellular uptake in U-87 spheroids (human glioblastoma cells) [53]. After 24 h, they observed a negligible penetration of unfunctionalized nanocarriers whereas the penetration of nanoparticles containing 25% of R8 was clearly deeper. In fact, 25% R8 nanoparticles were found within the first 5 cell layers of the spheroid, corresponding to a penetration depth of approximately 80 μm [53]. 

Apart from changing the size and charge of nanoparticles or modifying with cell ligands, it was found that spheroid penetration can be drastically improved upon enzymatic treatments. More specifically, the use of ECM-degrading enzymes, such as collagenase, turned out to strongly enhance the penetration depth of nanoparticles [34]. For 40 nm polystyrene beads, which normally reside at the periphery, collagenase treatment of SiHa spheroids (human cervical carcinoma cells) improved the penetration depth up to 11.6-fold [34]. Accordingly, equipping nanoparticles with ECM-degrading enzymes can be a good approach for enhancing the tumor permeability. The group of Cheng et al. has already shown the potential of such a system in vivo. In particular, they designed poly (ethylene glycol)-modified poly(glycolide-co-lactide) (PLGA-PEG) nanoparticles conjugated to hyaluronidase, an enzyme that degrades hyaluronic acid, one of the major ECM components. To prevent loss of enzyme function and reduced blood-circulation time, they added an extra PEG shell around the hyaluronidase. To evaluate the efficiency of their system, nanoparticles were intravenously injected into mice with 4T1 breast cancer tumors. They reported a significant increase in both nanoparticle diffusion in the ECM and tumor penetration of the hyaluronidase-PGLA-PEG nanoparticle compared to conventional PLGA-PEG nanoparticles. Additionally, hyaluronidase-PGLA-PEG nanoparticles loaded with doxorubicin could also efficiently inhibit the growth of 4T1 tumors [78]. The MCTS model can be used for further optimization of ECM-degrading functionalization.

### 2.2. Scaffold-Embedded 3D Models 

Although scaffold-free 3D models are suitable for studying the penetration of nanoparticles in solid tumors, the scaffold-free spheroid model lacks the so important tumor stroma, which the nanoparticles have to pass through in an in vivo setting. The tumor stroma consists of fibroblasts, endothelial cells, immune cells, and, importantly, ECM [79,80]. Scaffold-embedded models can be used to verify the influence of the ECM mimicking scaffolds on nanoparticle internalization. In more detail, in this section, we will discuss single cells and miniature tumors embedded in a scaffold, which exhibit a respective lower and a higher level of complexity. It is important to mention that MCTS can also be embedded in a scaffold. However, as the specific advantages of the spheroid model were discussed in the previous section and the influence of the ECM is evaluated better in single embedded cells, embedded MCTSs will not be discussed.

For embedded models, different types of scaffolds exist. They are often classified as natural or synthetic. The most used natural scaffolds are based on collagen, elastin, gelatin, hyaluronic acid polymer matrices or Matrigel^®^ [81]. The latter is a natural ECM, which is produced by mouse sarcoma cells and contains a mixture of ECM proteins including collagen, fibronectin and laminin. Intuitively, scaffolds containing naturally occurring polymers are the ones that more closely resemble the physiological ECM. On the other hand, since Matrigel^®^ is produced by mouse sarcoma cells, the protein composition ratio varies between batches and the biochemical and mechanical properties of the material can change. Therefore, the main disadvantage is associated to this batch-to-batch variation of the scaffold. Synthetic scaffolds have, however, the advantage of offering a full control of their properties. Polyglycolic acid (PGA), polylactic acid (PLA) and polyethylene glycol (PEG) are amongst the most popular scaffolds [82]. In the last years, there have been remarkable advances in the development of synthetic scaffolds for cell-culture applications [83]. One example is the new class of synthetic materials with biomimetic nonlinear mechanics developed by Kouwer et al. (polyisocyanide hydrogels, PIC) [84]. These hydrogels have an architecture and mechanical properties that closely resemble natural ECM and are uniquely suited as a 3D cell culture material [85,86,87]. 

#### 2.2.1. Scaffold-Embedded Cells 

**Figure 4:**
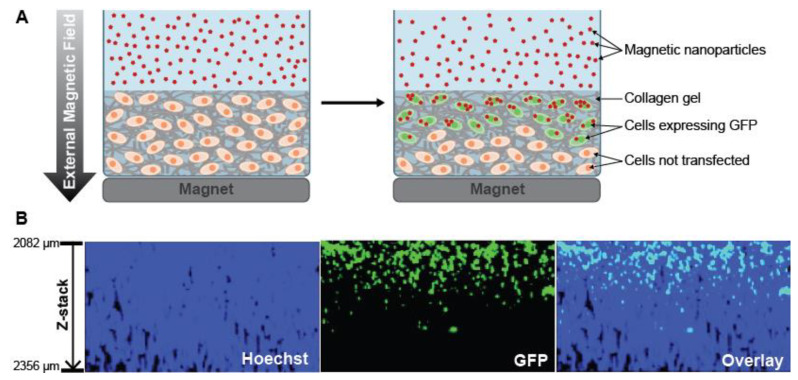
(**A**) Schematic representation of gene delivery by magnetic nanoparticles to 3D cell cultures seeded in a collagen matrix. (**B**) Z-stack image of 3D cell culture transfected with polyethyleneimine-coated superparamagnetic nanoparticles loaded with green fluorescent protein plasmid (PEI-coated SPMN/GFP) plasmid complexes for 3 h. Hoechst is shown in blue (first panel) while GFP transfected cells in green (second panel). Left hand scale: distance from the top of the culture. Adapted from Reference [90], with permission from American Chemical Society © 2020.

Just like the ECM in vivo, the presence of a scaffold in 3D models represents a physical hindrance for the nanoparticles as it implies that they have to pass through a dense polymer network in order to reach the cells. Scaffold-embedded models are, therefore, considered a good model to mimic nanoparticle-ECM diffusion and interactions, providing critical information on the relation between ECM and the accumulation of nanoparticles in the tumor in vivo. The fiber network of the scaffold can be a limiting factor on the internalization rate, as particle diffusion is sterically blocked [34,88,89,90]. In the work of Zhang et al., the efficiency of several commercially available transfection vehicles was investigated using cells embedded in a collagen matrix (e.g., Lipofectamine, FuGene HD, JetPEI, Polymag, etc.). All the transfection reagents tested resulted in a transfection efficiency below 1%, possibly as a result of poor diffusion of the lipid/nucleic acid complexes though the collagen matrix. The transfection efficiency of nanoparticle-mediated DNA delivery was also evaluated. The authors compared commercial magnetic nanoparticles used for transfection (PolyMag, 250 nm) and their home-synthesized, smaller, polyethyleneimine-coated superparamagnetic nanoparticles (SPMN, 35–55 nm depending on the amount of nucleic acid bound). Despite the supplementary application of an external magnetic force to drive the magnetic nanoparticles through the matrix, the commercial magnetic nanoparticles also yield a low transfection efficiency of less than 5%. A higher transfection efficiency could be achieved with the developed PEI-coated SPMNs. Nevertheless, as a result of the incomplete diffusion of the particles across the entire matrix, the maximum penetration depth of transfected cells was limited to 2.1–2.3 mm (after 3 h of exposure to a magnetic field (Figure 4) [90].

**Figure 5:**
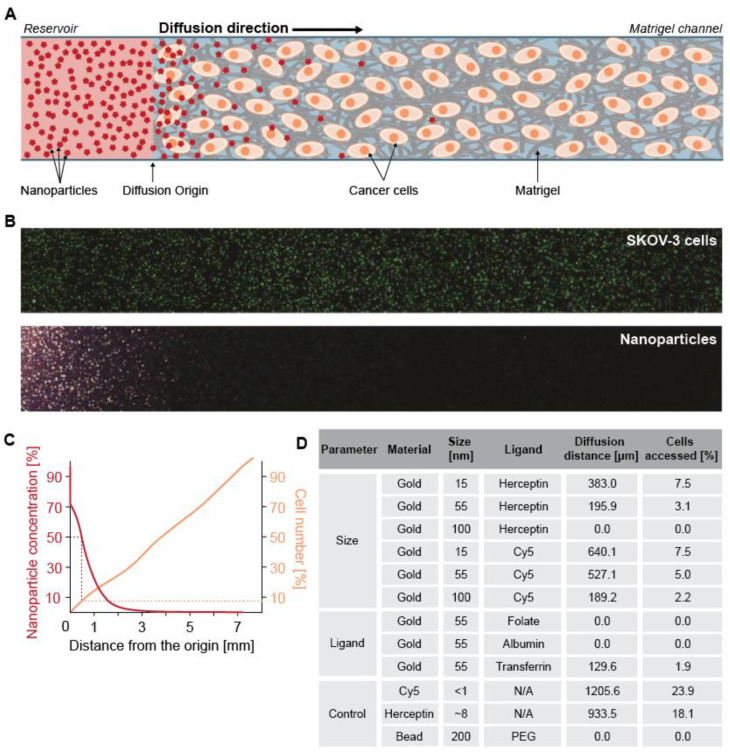
(**A**) Scheme of nanoparticle diffusion in 3D SKOV-3 cells (ovarian cancer cell line) embedded in Matrigel^®^ that shows impeded access of nanoparticles to the SKOV-3 cancer cells due to the Matrigel^®^ presence. (**B**) Confocal images of SKOV-3 cells and nanoparticles in the Matrigel^®^. (**C**) Quantification of nanoparticle diffusion distance and the related percentage of SKOV-3 cells accessed by the nanoparticles. The diffusion distance was indicated as 50% of the initial nanoparticle concentration away from the reservoir (orange line). The corresponding distance also reflected the percentage of cells the nanoparticles had access to (orange line). (**D**) The average values of the diffusion distance and the percentage of accessed cancer cells the nanoparticles for other nanoparticles with various design parameters. Adapted from reference [91], with permission from American Chemical Society © 2020.

In a more recent study, SKOV-3 cancer cells were evenly encapsulated in a Matrigel^®^ scaffold and 17 different types of nanoparticles were supplemented from a starting reservoir (Figure 5) [91]. The different nanoparticles ranged from 15 to 200 nm in diameter, were decorated/not decorated with targeting agents, made from different materials and supplied/not supplied with a protein corona (layer of serum proteins adsorbed on the nanoparticle surface [92,93]). The authors established that, independently of the three different Matrigel^®^ concentrations tested (12.5, 50 and 70%) and the type of nanoparticle used, the nanoparticles reached less than 8% of the cells (Figure 5). These results indicate that the presence of the Matrigel^®^ represents a major hindrance to the diffusion of nanoparticles and suggest that in vivo the presence of ECM will drastically limit their accessibility to a tumor. Additionally, the authors proposed that not only the ECM but also the presence of tumor-associated macrophages (TAMs), another component of the tumor stroma, can have a critical effect on nanoparticle internalization by cancer cells. The phagocytic ability of macrophages, combined with their proximity to the tumor, results in TAMs acting as uptake competitors for cancer cells, having a higher rate of nanoparticle internalization. As a proof of concept, HER-2-targeted nanoparticles were administered into a mouse breast cancer model. Less than 14 out of 1 million nanoparticles intravenously injected, reached the cancer cells. This result was associated with both entrapment in the ECM and nanoparticle internalization by non-cancer cells in the stroma [91]. This hypothesis can be evaluated using co-cultured spheroids. For instance, in the work of Tevis et al., they established two co-culture models, one in which macrophages are seeded in the collagen matrix surrounding a breast cancer spheroid, resembling the TAMs in the tumor stroma [31]. Incorporation of macrophages within a breast cancer spheroid increased the resistance to paclitaxel. To the best of our knowledge, there are no studies on how the presence of TAMs influences the cellular uptake of nanoparticles in (hetero) spheroids.

**Figure 6:**
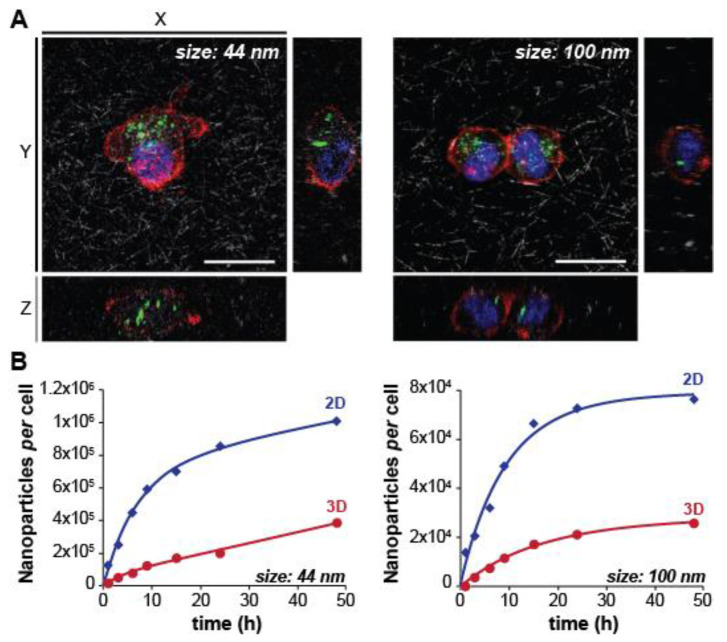
(**A**) Confocal microscopy maximum projection of Z-stack sections, obtained by confocal microscopy, of HT1080 cells incubated with 44 nm and 100 nm nanoparticles (NPs) for 24 h in 3D collagen matrix. Cell nuclei are shown in blue, actin microfilaments in red, NPs in green and collagen fibers in grey. Scale bar: 10 µm. (**B**) Uptake kinetics of 44 nm and 100 nm NPs by human epidermal fibroblasts (HDF) cells during continuous exposure to the nanoparticles, as determined by spectrophotometric analysis. Data points and error bars represent the mean and standard deviation over three replicas. Adapted from Reference [88], with permission from Elsevier B.V. © 2020.

Recently, the differential nanoparticle uptake in scaffold-embedded cells was evaluated at a single cell level. Belli et al. compared the uptake of 44 and 100 nm polystyrene nanoparticles by cells cultured on plastic and within a collagen matrix [88]. Although the overall uptake of 44 nm nanoparticles was higher, there was a drastic decrease in internalization rate in 3D for both 44 and 100 nm particles (Figure 6). Apart from the known physical hindrance of the collagen network, the authors highlighted the possible involvement of the cytoskeleton structure on this outcome [88]. It is well-known that the cell’ shape in a 3D matrix is very different from the morphology of cells cultured on plastic/hard surfaces. On the latter, the contact surface between the membrane and the surface is very large, while in a scaffold, the cell membrane interacts with the ECM-mimicking scaffold at discrete locations [94]. This difference results in a stretched morphology with a well-defined cytoskeleton for cells grown on 2D substrates, whereas, in 3D scaffolds, the cytoskeleton typically re-organizes [95,96,97]. Note that, despite its difference with 2D culture systems, the morphology of cells embedded in a 3D scaffold resembles more the in vivo physiology [98,99]. 

Besides affecting the cellular structure, the cytoskeleton is known to play a role in many cellular processes, namely endocytosis and vesicle trafficking [100,101]. Different reports suggest that differences in the cytoskeleton structure of cells grown in stiff and soft substrates correlate with a significant decrease of nanoparticle internalization [88,102]. Other cellular processes affected by a cytoskeletal reorganization include proliferation, differentiation and even drug response/resistance in cancer [95,103,104,105,106,107]. Moreover, the spatial organization of surface receptors can be altered in a 3D compared to 2D cellular configuration, which may alter uptake efficiency of receptor targeted drugs/nanocarriers [13,108]. 

The physical barrier and the biological changes induced by the ECM present in vivo can be elucidated in vitro using scaffold-embedded 3D model systems. Physical hindrance caused by the dense ECM is a limiting factor in nanomedicine delivery and knowledge on the behavior of nanoparticles in the ECM is poor. Similarly, there is still a lot to be discovered about the biological changes induced by the substrate/environment and how these influence the cellular uptake of nanoparticles (i.e., receptor distribution, endocytosis, cytoskeleton organization). 

#### 2.2.2. Human-Derived Cancer Organoids 

Scaffold-embedded single cells or spheroids mimic some of the characteristics of solid tumors but lack the physiological relevance of the organ of origin. Nowadays, researchers are able to grow miniature versions of organs also known as ‘organoids.’ When stem cells are embedded in supporting extracellular matrices (Matrigel^®^) and cultured with Wingless-type MMTV integration site (WNT) signaling agonists, they self-organize into mini organ-like structures [109]. Fully grown organoids can be passaged and regrown for months, making them long-term expandable. For example, a protocol to grow and maintain organoids derived from human endometrial cancer was recently described by Boretto et al. [110]. This publication is a good example of how to create organoids from cells of cancer patient biopsies and highlights the potential of these in vitro models to develop personalized medicine [109,110,111,112,113]. Recently, Driehuis et al. reported an overview of the different protocols for the generation of organoids from various cancer types, including a protocol to test the sensitivity of patient-derived organoids to specific cancer therapies [114]. The patient specificity makes organoids extremely popular in personalized medicine and drug screening tests. Immense breakthroughs have already been made by using these models in treatment response prediction tests [112,115,116,117]. Cancer organoids (or tumoroids), recapitulate the in vivo tissue heterogeneity, including the presence of a stem cell population [118]. Therefore, they represent an essential model for cancer stem cell research as well. The cancer stem cells subpopulation is nowadays thought to be the main culprit that leads to metastasis, as well as disease recurrence due to its high resistance to treatment. 

In nanomedicine, these models can be used to study ECM-nanoparticle interactions, nanoparticle diffusion in the ECM, nanoparticle-organoid encounter and nanoparticle interactions with the heterogenic organoid environment. Importantly, this model can be used to evaluate which cell types internalize the nanoparticles and which do not. Despite its potential, the reports using human cancer organoids to evaluate the therapeutic effect of nanomedicines are scarce to non-existing at this moment. However, there are records of nanoparticles being implemented in human organoid models for purposes other than drug delivery, namely cytotoxicity tests, mechanical modulation and (fluorescence) imaging. For example, in the recent study of Park et al., the toxicity of silicon dioxide (SiO_2_) and titanium dioxide (TiO_2_), which are food additives serving as an anti-clumping agent, were evaluated in human colon organoids. The colon organoids were cultured in 60% Matrigel^®^ and 40% customized organoid medium. Both types of nanoparticles were incubated with colon organoids for 48 h. After incubation, a live/dead assay revealed that increasing concentrations of both nanoparticles induced elevated cell death with IC_50_ values of 0.3 mM and 12.5 mM for SiO_2_ and TiO_2_, respectively [119]. Another example is the study of Bergenheim et al., where fluorescently-labeled nanoparticles are used as a tool to label colon organoids for tracing transplanted cells. The colon organoids were cultured in 40% standard culture medium and 60% growth factor-reduced Matrigel^®^. The labeling efficiency of both a quantum dot solution and 150.6 nm PLGA nanoparticles loaded with a BOPIDY-FL dye were tested. Quantum dots were incubated for 1 h, whereas PGLA nanoparticles were incubated for 4 to 6 h, 24 h or mixed into the diluted Matrigel^®^ solution to minimize their diffusion distance to the organoids. Unfortunately, labeling of the whole organoid could not be achieved since both PLGA nanoparticles and quantum dots became associated to the membrane rather than being internalized into the cells. The authors speculate that nanoparticle uptake is hindered by the inherent properties of organoids, in which the basal surface of the cells in the periphery faces the surrounding Matrigel^®^ and the apical side points towards the lumen. As endocytosis occurs primarily on the apical site, this polarization might reduce the uptake of nanoparticles. In addition, in agreement with the reports mentioned in Section 2.2.1 [90,91], the authors state that the Matrigel^®^ itself acts as a physical barrier for nanoparticle diffusion [120]. 

Although cancer organoids already exemplify one of the most high-end 3D cell models available, the tumor microenvironment can be mimicked by using hybrid cancer organoids, where cancer cells are co-cultured with fibroblasts in an ECM-mimicking scaffold [121]. While these models strive towards a more realistic view on the complex in vivo physiology of the tumor tissue, their application in nanomedicine development remains limited. This can be attributed either to their implementation at the research labs or to the lack of a proper characterization. Organoids have been developed very recently (little over a decade ago), and consequently, the know-how in this field is relatively limited. In order to develop a long term expandable organoid line from a particular cancer biopsy, researchers face a lot of trial-and-error, especially in optimizing the growth medium. This medium should contain the perfect cocktail of growth factors and proteins for the cancer cells from a specific biopsy to grow into organoids. Moreover, once the organoids are established, expanding the culture remains challenging, often requiring further optimization. Taken together, the process of starting up an organoid culture from scratch can take months to years. In addition to extensive expertise needed to grow quality organoids, the technique itself is also costly. The specific growth factors and proteins required for the medium cocktail are usually very expensive. It is worth to mention that this cocktail is not required when growing spheroids. Nevertheless, organoids remains one of the models with the highest level of physiological relevance, as they are derived from patients directly and present close similarities to the tumor in vivo, including drug response. Furthermore, it is the only model that allows the investigation of all the cell types present in a given organ. In line of this, at this moment, organoids are a hot-topic in the field of personalized medicine [112,115,116,117]. 

### 2.3. Microfluidic Platforms

All the previously discussed models still lack one crucial aspect when it comes to mimicking the in vivo physiology: the fluid dynamics. The delivery and accumulation of nanomedicines in tumor tissues in vivo remains the major obstacle in the development of better drug delivery systems. A better understanding of how the nanoparticles diffuse in the blood vessels to reach their target tissue and how they distribute at the cellular level is crucial to improve the biological performance of nanomedicines. Microfluidic devices offer a customizable platform to investigate complex (multi)cellular structures under controllable flow conditions. Importantly, the absence of fluid flow in conventional 2D cell culture systems can influence the results obtained. For instance, Fede and coworkers evaluated the uptake and toxicity of gold nanoparticles in human endothelial cells under both static and flow conditions [122]. They discovered that the internalization of gold nanoparticles was significantly lower when administered under flow, in a microfluidic channel, than when they were administered under conventional static conditions (where the nanoparticles settled on top of the cells). More specifically, from the 10^12^ NP/mL administered in both conditions, 29.2% and 0.17% were internalized by the cells in the static and flow condition, respectively. Accordingly, they found that the cell toxicity under flow conditions was around 20% lower compared to the static condition. 

**Figure 7:**
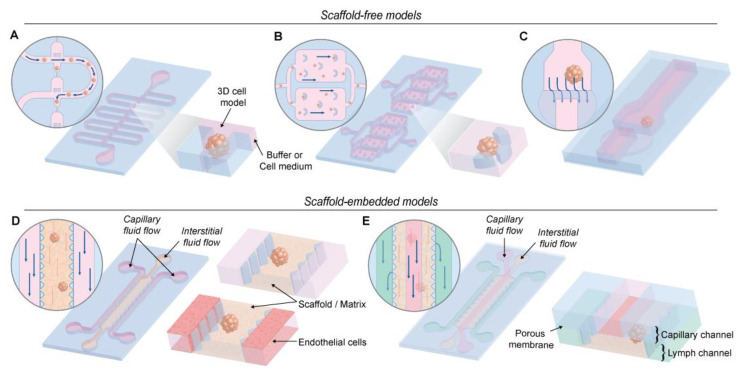
Schematic representation different microfluidic devices used. (**A**) A device containing a sinuous microchannel each of which contains five microwells for sedimentation trapping of the loaded microtissues or spheroids [127]. (**B**) A device containing permanent U-shaped microstructures. The flow of culture medium towards the microstructure pushes the cells inside [125]. (**C**) A two-layer microfluidic chip. The spheroid is immobilized in the higher part of the channel before the dam, where the height decreases from 250 to 25 µm [129]. (**D**) These devices consist of 2 media channels engulfing an extended, central region containing the scaffold/matrix. The devices contain an array of trapezoidal posts that cage the matrix solution into well-defined regions with uniform surface interface. The side channels can be perfused with endothelial cells to simulate the endothelial wall [130,132]. (**E**) Microfluidic platform with three types of channels - capillary, interstitial and lymphatic channels (noted with red, yellow and red colors). These channels are independently pressurized to mimic the elevated interstitial fluid pressure and are configured in a 3D structure by stacking two polydimethylsiloxane (PDMS) layers of microchannels with a porous membrane sandwiched between the layers [123].

In 3D cell models, particle sedimentation reduces the cell-particle contact time, which can hamper the use of these models to test the therapeutic efficiency of nanomedicines. To counteract this phenomenon, in recent years, microfluidic devices have attracted much attention, especially in drug testing [123]. Concerning nanoparticle testing on 3D cancer-mimicking systems, different microfluidic platforms have been developed. As depicted in Figure 7, the microfluidic devices can be categorized into two groups: scaffold-free and scaffold-embedded models. In the second category, the flow might be applied directly on the scaffold or on an endothelial layer (or other cells) grown adjacent to the scaffold-embedded spheroid.

The simplest microfluidic platform consists of a 3D cell aggregate (spheroid), entrapped in a microfluidic chamber that is placed at physiological flow conditions [124,125,126,127] (Figure 7A,B). Accordingly, nanoparticles can be administered via this flow of media, with the flow velocity being adjusted to the one found in human capillaries. Huang and coworkers used the device depicted in Figure 7B to study the penetration of 100 nm polystyrene nanoparticles with a positive or negative surface charge in spheroids in static conditions or under physiological flow, with or without serum proteins in the culture medium [125]. When nanoparticles are in a medium that contains proteins, the interaction between proteins and the surface of the nanoparticles induces the formation of a protein layer surrounding the particle, the so-called ‘protein corona.’ The characteristics of this layer (e.g., thickness) depend on the properties of the nanoparticles [92,93]. This system enabled the investigation of the effect of the charge of the nanoparticles, the presence of a protein corona as well as fluid flow, on their penetration in spheroids. In the absence of a protein corona, negatively charged nanoparticles showed a higher accumulation (at the periphery) and enhanced penetration. When medium containing serum proteins was used, positively charged particles accumulated less at the periphery of the spheroid but showed an increased penetration. The authors suggest that it is caused by the presence of a protein corona, which preferentially forms on positively charged particles and is known to decrease cell binding potential of the particles. The presence of a flow flushed away peripheral nanoparticles, especially those ones with a protein corona (loosely attached). However, the flow also promoted deeper penetration into the spheroids. In summary, the deepest penetration in the spheroid was achieved with negatively charged nanoparticles, without a protein corona, under a physiological flow [125]. In a similar configuration, Toley and coworkers trapped colon adenocarcinoma spheroids (LS174T cells) in a microfluidic channel and perfused them with medium containing either doxorubicin or the commercial Doxil^®^ (liposome encapsulating doxorubicin). Taking advantage of the inherent fluorescence of doxorubicin, fluorescence images of the cancer tissue were acquired to monitor the uptake and consequent clearance of the two drug formulations. A high uptake into the cancer tissue was observed for both doxorubicin and Doxil^®^, however Doxil^®^ was not retained in the tissue (clearance after 8 h). The longer retention of doxorubicin suggests a stronger binding to the cancer tissue and DNA intercalation, whereas for Doxil^®^, the release of doxorubicin inside the cancer tissue was minimal [128]. 

**Figure 8:**
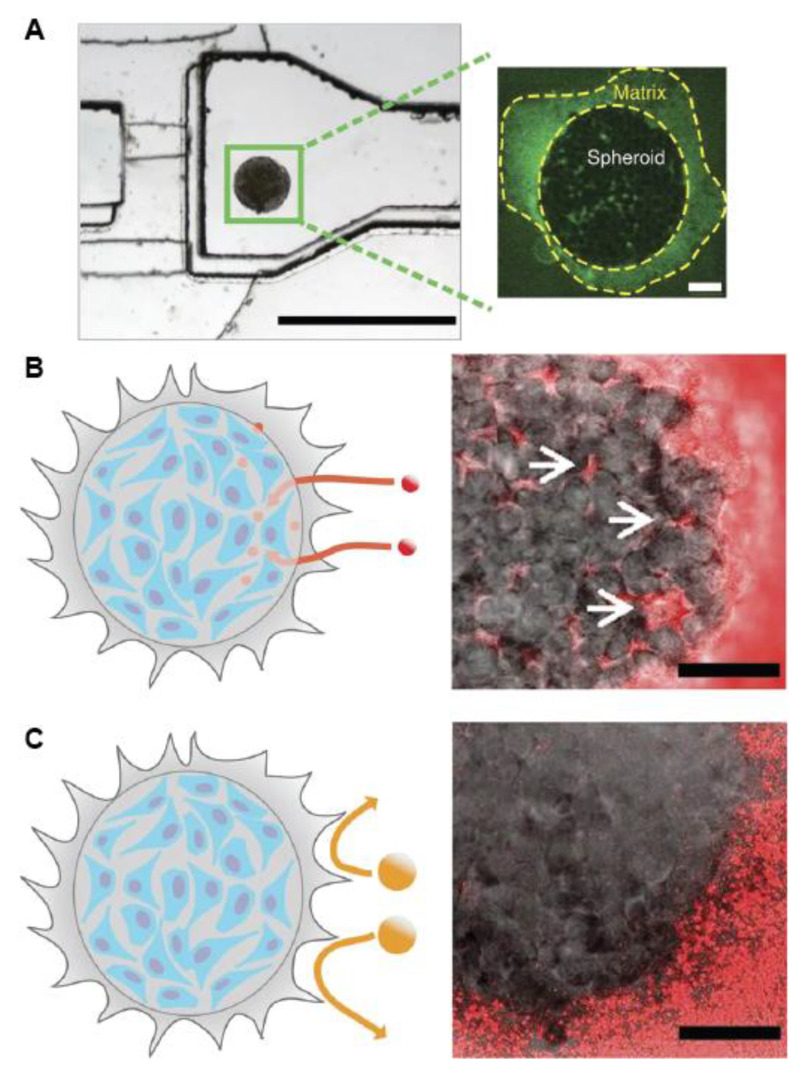
(**A**) Image of the microfluidic device demonstrating the channel dimensions and the immobilized spheroid in the imaging chamber. Scale bar left panel = 1000 μm, right panel = 100 μm. (**B**) Scheme (**left**) and image (**right**) of 40 nm fluorescent poly(ethyleneglycol)-coated nanoparticles (PEG-NPs) administered for 1 h at 50 μL h^−^^1^ penetrating the spheroid and accumulating in the interstitial spaces (arrows). Scale bar = 100 μm (**C**) Schematic (**left**) and image (**right**) of 110 nm fluorescent PEG-NPs administered for 1 h at 50 μL h^−^^1^ being not penetrating the spheroid. Scale bar = 100 μm. Adapted from Reference [129], with permission from Nature © 2020.

To evaluate the influence of the ECM surrounding the spheroid, Albanese et al. used spheroids grown in medium containing 2.5% of Matrigel^®^ [129]. After 3 days of culture, the spheroids were immobilized in the channel of a polydimethylsiloxane (PDMS) microfluidic system, where different sizes of PEGylated gold nanoparticles were administered (Figure 8B,C). The fluid flow was adapted to be similar to either the blood flow in the capillary vessels or the interstitial flow inside the tumor. The results obtained in this work established that only the small nanoparticles (hydrodynamic diameter of 40 nm and 70 nm) can significantly reach the interstitial tumor space. Since PEG coating prevents interaction with ECM proteins, PEGylated nanoparticles in the interstitial tumor space could be flushed away when washing the chamber with a clear solution. When the 40 nm gold nanoparticles were functionalized with transferrin, which interacts with the cell by receptor binding, accumulation in the spheroid was increased up to 15-fold. In this case, washing the chamber did not flush the targeted nanoparticles away from the spheroid, indicating specific receptor binding. Another approach to investigate the influence of the ECM in the diffusion of nanoparticles is to fill a chamber with a scaffold (Figure 7D,E). Using the microfluidic device depicted in Figure 7D [130]. Schuerle et al. have recently shown that the penetration of nanoparticles in collagen gels can be increased by adding micropropellers powered by a rotating magnetic field [131]. The authors showed that the use of such micropropellers increased the nanoparticle transport into the adjacent collagen matrix by enhancing local fluid convection. 

To recreate vascularization, scaffold-embedded spheroids can be immobilized in close proximity to a layer of endothelial cells [132,133]. In nanomedicine, this system can be of particular interest to investigate how nanoparticles overcome (or not) the endothelial barrier. In vivo studies showed that particles with a diameter between 20 nm and 200 nm accumulate in the tumor tissue. The most accepted hypothesis is that this accumulation results from the presence of leaky vascularization (impaired tight endothelial junctions) around the tumor, which is absent in healthy tissue. This phenomenon is referred to as the enhanced permeability and retention (EPR) effect [134,135]. Although the concept of EPR has been generally accepted over a decade now, more and more controversy around the topic has arisen [136,137,138]. In this context, vessel-on-a-chip devices represent the ultimate in vitro model to perform in-depth research on this ongoing argument. The combination of flow, an endothelial wall, ECM and embedded MTCSs, offers a good simulation of the barriers that nanocarriers have to overcome in vivo. Using this approach, Feiner Gracia et al. monitored the stability and extravasation of self-assembled micelles using spectral confocal microscopy [132]. The formation of leaky vasculature could be detected in the regions where cancer cells were in close proximity to the endothelial layer. Moreover, the authors could evaluate the performance and stability of the micelles in each of the barriers. While some micelles crossed the endothelial layer as assembled micelles, others were disassembled. Interestingly, one type of micelle lost their stability close to the cancer spheroid but all the micelles studied showed low penetration into the spheroid. In another study performed by Agarwal et al., a 3D vascularized tumor network was designed by combining micro-tumors of MCF-7 cells (cancer cell spheroids with a diameter below 200 μm) with stromal cells (endothelial cells and adipose-derived stem cells) in a collagen hydrogel in a perfusion chamber. Once the microfluidic system was fully characterized, the authors evaluated the effect of vascularization on the cancer resistance to free doxorubicin and doxorubicin-loaded nanoparticles (lipid nanoparticles with a fullerene core embedded in a matrix of doxorubicin and indocyanine green-encapsulated mesoporous silica nanoparticles, approximately 60 nm diameter). Based on viability assays, they report that 3D vascularized tumors are 4.7 times more resistant to doxorubicin than avascular tumors and even 139.5 times more resistant than 2D cultures. Remarkably, doxorubicin-encapsulated nanoparticles were more effective than free doxorubicin in the 3D vascularized tumor, dropping the IC50 to 16 μg/mL [139]. 

The majority of vascularized microfluidics lacks an important component of the microvascular system: the lymphatic endothelial cells. The lymphatic system collects fluid and proteins from the interstitial space, returning them to the blood circulation and is responsible for maintaining the interstitial flow. Ozcelikkale et al. developed a microfluidic chip where three types of channels—capillary, interstitial and lymphatic channels—are independently pressurized to mimic the elevated interstitial fluid pressure at the tumor microenvironment (Figure 7E) [140]. The authors used this device to characterize the delivery and efficacy of free doxorubicin and doxorubicin-encapsulated hyaluronic acid (HA) nanoparticles, in two different breast cancer cell lines (MCF-7 and MDA-MB-231). Doxorubicin accumulated and penetrated similarly in both cell lines. HA nanoparticles accumulated more in MDA-MB-231 than MCF-7, most likely due to the higher expression of CD44 (HA receptor). In agreement with results obtained using scaffold-free spheroids, the larger size of the nanoparticles limited their penetration in the cell aggregates. Interestingly, both cell lines cultured on the microfluidic chip showed increased resistance to the drug compared to 2D culture systems.

To better resemble the physiological microenvironment of cancer in the human body, the microfluidic devices (so-called tumor-on-chip) can, in addition to the cancer cells, endothelial cells and ECM, also incorporate other cell types found in the tumor stroma, namely fibroblasts and immune cells [141]. These cells can be encapsulated in the ECM-mimicking scaffold or within the spheroid (forming heterospheroids). In this way, microfluidic devices can harbor all aspects present in an authentic physiological setting. In recent years, different devices have been developed, aiming to mimic the physiology of different organs [142]. It is now possible to use an ‘organ-on-chip’ where different compartments containing cells from different organs are present and connected based on their biological sequence to evaluate how different drugs affect different organs [143]. An example is the work of Esch et al., where the gastrointestinal (GI) tract and liver were simulated on a chip. In this model, the researchers mimic the oral uptake of 50 nm carboxylated polystyrene nanoparticles and validate whether they cause tissue damage. The GI tract was represented by a co-culture of enterocytes, Caco-2 and mucin-producing cells, TH29-MTX, whereas the liver was recreated by HepG2/C3A cells, all together in one microfluidic setup. Fluorescence imaging shows that the GI can function as a barrier for 50 nm polystyrene nanoparticles, since the overall majority is still at the apical side of the cell layer. However, nanoparticles that crossed the GI could induce liver damage, as suggested by an increase of the aminotransferase (AST) release 24 h after nanoparticle administration [144]. 

Although the potential of these microfluidic platforms to mimic the situation in vivo is undeniable, the highly challenging fabrication of these elaborated devices has to be taken into account. In addition to the design of the chip, the models requires careful characterization before being used.

## 3. Characterization and Biological Assays for 3D Models 

Compared to 2D monolayer cultures, 3D culture models are more complex and often contain multiple cell types (e.g., organoid and some microfluidic models). Furthermore, the presence of a third dimension and more cell-cell interactions introduce challenges in the characterization of 3D cell culture systems and require significant adaptations in the biological assays currently used. One of the most popular techniques used in characterization and biological assays is fluorescence microscopy. While the majority of the protocols used for fluorescence assays using 2D culture systems are well established, for 3D cell models sample preparation still requires optimization and assays often required prior validation. For instance, in scaffold-embedded 3D models the presence of a scaffold can act as a physical barrier for diffusion of molecules or proteins towards their target. On the other hand, the (bio) chemical properties of the scaffold itself might interfere with the compounds used (e.g., antibodies used for immunofluorescence). This might affect the data obtained from biological assays involving chemicals, drug responses curves and, immunofluorescence images [145].

To reduce the effect of the physical barrier imposed by the scaffold, protocols are often optimized with prolonged incubation times of specific reagents. To this end, O’Rourke et al. described a detailed protocol for antibody-staining of Matrigel^®^-embedded mouse intestinal organoids. In this procedure, the permeabilization was improved by increasing the concentration of the detergent used (Triton X-100) [146]. Next to the scaffold, the dense multilayered nature of the spheroids and some organoid models might hamper a uniform diffusion of the reagents. Therefore, even in scaffold-free 3D cell models, longer incubation times of antibodies/reagents or prolonged washing steps are often required. In 2010, Weiswald et al. already acknowledged the problem of poor antibody penetration into spheroids and developed an optimized protocol for the fluorescence staining of different antigens in HT29 and CT320X6 cells [147]. In agreement with the report of O’Rourke, the procedure included a longer incubation with the fixative and increased permeabilization by using a higher detergent concentration (3 h, 1% Triton X-100). 

Fluorescence imaging of 3D cell models is also hindered by the reduced penetration of light in the multicellular structures. Improved of 3D cell culture systems can be obtained by optical clearing of the sample. This procedure reduces light scattering and allows deeper light penetration during fluorescence-based measurements. Several groups have published successful protocols for the optical clearing of organoids and spheroids, drastically improving the results obtained by fluorescence microscopy [148,149]. Among them, the Boutin et al. developed a procedure for high-throughput optical clearing of spheroids, followed by imaging and a high-throughput image analysis, using 3D segmentation of the nuclei [150]. Another technique that holds great potential for imaging 3D cultures is expansion microscopy. In this approach, following the embedding of the sample in an expandable hydrogel, lipids are removed and the sample is expanded approximately 4.5 times. This physical expansion of the sample enables super-resolution imaging on a conventional diffraction limited microscope. As such, expansion microscopy combines both optical clearing and higher spatial resolution [151]. Recently, Edwards and coworkers performed expansion on several tumor spheroids and organoids, stained with various antibodies (anti-tubulin, anti-p-histone, etc.), small organic dyes (e.g., DAPI) and fluorescent proteins (green fluorescent protein, yellow fluorescent protein, etc.). Imaging was performed on a light sheet microscopy set-up. Their results showed increased spatial resolution, isotropic expansion and improved signal to background ratio on the measured samples [152]. 

Aside from the required optimization of sample preparation methods, imaging 3D cell models with sub-cellular resolution is far from trivial. Standard confocal microscopes are not the most suitable to image these models. This limitation is due to the short working distance of most objectives, poor penetration depth of the excitation light and the light scattering nature of thicker samples. To circumvent these issues, researchers can use thin slides of the sample (e.g., obtained using cryosectioning). However, the use of sections often hinders the 3D reconstruction of the sample, which is crucial when investigating 3D models, and excludes the possibility for live cell imaging. An alternative approach is to use more advanced microscopy techniques, such as multi-photon laser scanning microscopy or light sheet microscopy. Multi-photon microscopy involves the use of a confocal setup equipped with a multi-photon excitation laser. Multi-photon excitation uses red-shifted light which results in deeper light penetration into the sample, and, consequently, a more uniform excitation on thicker samples. Light sheet microscopy [153] and more recently, single objective light sheet microscopy [154], have gained much interest as these techniques enable fast, live cell imaging of thick 3D samples. Using these novel imaging methods, information on dynamic processes in 3D cell models have been obtained. By using light-sheet microscopy, Alladin and coworkers visualized the breast tumorigenesis process in murine organoids over time. In this model, a few tumorigenic cells were introduced in a primary mammary epithelial organoid. These single cells could be followed overtime, as they were transforming neighboring cells into tumorigenic cells [155].

## 4. Summary and Future Directions 

Typically, in nanomedicine research, the efficiency is measured on 2D culture systems and, when positive results are obtained, research is often directly translated to in vivo animal models. It is important to mention that while researchers can nowadays mimic important characteristics of the cancer physiology using in vitro 3D tumor models, even the most advanced 3D cancer models cannot fully mimic the tumor physiology and, therefore, they cannot fully replace animal models in drug delivery research [156]. Nevertheless, a mouse model is not always the most physiologically relevant model to investigate a human disease. In this sense, highly developed 3D culture models such as spheroids, organoids and organ-on-chips might serve as better in vitro models. Spheroids in particular, because of their easy manufacturing process, allow high-throughput screening of pharmacological responses, whereas organoids, despite the more complex manipulation, are actively used to screen for patient specific drug responses in personalized medicine. Both high-throughput screening and the ability to perform personalized medicine are more difficult to achieve in a preclinical animal model (mostly because of the long time required to generate such a model). With the arrival of the organ-on-chip platforms, introducing vascular perfusion, tissue-tissue interaction and the presence of different cell types in one model, the possibility arises to study drug response in a more biologically relevant system, revealing drug (nanocarrier) interactions at the tissue and single cell level. The miniaturized size, a characteristic shared amongst all the 3D in vitro models discussed here, enables the study of the behavior of nanoparticles in a more selective and detailed manner. The mechanistic insights achieved can be easily overlooked in animal models, where in vivo imaging or tumor resection are the most commonly used methods to analyze the effect of specific drugs or nanocarriers. 

In this review, we compared the 3D models currently available, pinpointed their pros and cons and described to what extent each model can contribute to nanomedicine development (Table 2). More specifically, nanoparticle accumulation and penetration in solid tumors can be investigated in depth using scaffold-free spheroid models. Using this 3D model, different research groups have shown that smaller particles can penetrate deeper in 3D multicellular structures. However, for improved circulation times, interactions with hepatocytes and renal clearance limit the nanoparticle size to 50 nm and 10 nm, respectively. This has led researchers to develop size-switching nanoparticles. Typically, these particles have an original diameter of 50–100 nm and, upon an external trigger, release smaller particles. Although this correlation between nanoparticle size and penetration depth is generally accepted, there are contradictory results from the influence of nanoparticle surface charge on spheroid penetration. Most reports suggest that negatively charged particles will penetrate further but it depends on the specific functional group exposed on the surface of the particle. In fact, functionalization with specific ligands or cell-penetrating peptides is also used to enhance the penetration of the particles in MCTS. 

To investigate ECM-nanoparticle interactions and nanoparticle diffusion through the ECM, scaffold-embedded models can be used. Similar to the so-called ECM barrier in vivo, the presence of the scaffold often introduces difficulties regarding nanoparticle diffusion. Both the works of Zhang et al. [90] and Dai et al. [91] individually show the influence of the physical barrier introduced by the scaffold, which resulted in a low percentage of embedded cells containing nanoparticles. In a more complex 3D model, colon organoids were embedded in Matrigel^®^ and treated with SiO_2_ and TiO_2_ nanoparticles to check their cytotoxicity by live/dead staining, revealing increasing cell death with increasing concentrations of these two food additives.

In vitro 3D cellular systems can be rendered even more realistic by mimicking the vascular systems. By using microfluidic devices, researchers are able to mimic closely an in vivo setting with the possibility to perform nanoparticle studies and imaging at high resolution. These models are especially relevant to address the controversial EPR-effect.

While the examples discussed in this review show the importance of 3D culture systems in obtaining crucial knowledge on the behavior of nanoparticles, the use of these 3D models in nanomedicine research is still not a standard practice. Nowadays, the majority of the methods used for preparing 3D cell culture systems are well-established (especially for the MCTS model). However, most biological assays and characterization methods used are still optimized for 2D culture models. In fact, their translation into 3D cell models poses one of the major challenges for the extensive implementation of 3D cancer models. Fluorescence microscopy has drastically contributed to unravelling biological processes and characterizing cell assemblies in the last decades. However, its application on 3D cell culture systems is still challenging due to demanding sample handling and preparation. More specifically, staining protocols for 3D models must be optimized and samples often require extra manipulations, such as optical clearing. Another challenge in imaging these models is related to the setup used. For instance, standard confocal microscopy techniques are often not suitable to image 3D cell models. In the last years, advances in imaging techniques have been made, significantly improving the imaging quality of 3D samples, while enabling live imaging. Examples are multi-photon laser scanning microscopy and (single objective) light-sheet microscopy. 

In addition to the technical challenges, there are also biological and physical issues are not yet fully understood. Especially nanoparticle and ECM interactions are still a “tough nut to crack.” The diffusion of nanoparticles through the scaffold can be improved by tuning their size and charge, however, there is only a handful of reports addressing this optimization step. Other overlooked aspects included the effect of the scaffold in the cell biology (receptor distribution, cytoskeleton organization) and the cellular heterogeneity. Implementation of the 3D culture systems can shed light into the reasons behind the different efficiency of nanomedicine in in vitro and in vivo models. More important, 3D models can build a fundamental bridge between in vitro and in vivo studies, ultimately paving the way for nanoparticle translation to in vivo and clinical stages.

## Figures and Tables

**Table 1:** Overview of nanoparticle studies using the multicellular tumor spheroid (MCTS) model.

Nanoparticle	Diameter (nm)	Cell Type ^1^	Cancer Type	Strategy	Parameter(s) Studied	Ref.
Lipid nanoparticle (Lipidots)	50, 120	CALL-33	Oral tongue cancer	n/a	Penetration Viability	[36]
Polystyrene	30, 50, 100	HCT116	Colorectal cancer	n/a	Penetration Surface charge dependence	[37]
Dendrimer	2 and 8	KB	Epidermal	n/a	Penetration Surface charge dependence	[38]
AuNPs	50 and 100	MCF-7	Breast cancer	n/a	Penetration	[39]
AuNPs	2, 6 and 15	MCF-7	Breast cancer	n/a	Penetration	[40]
Silica	30, 100	4T1 and 3T3 co-culture	Breast cancer	n/a	Penetration influenced by tumor stroma	[41]
PGLA	200	4T1 and 3T3 co-culture	Breast cancer	n/a	Penetration influenced by tumor stroma	[41]
Au nanorod	55 × 15	MCF-7	Breast cancer	n/a	Penetration Surface charge dependence	[42]
				**ECM destabilization**		
Polystyrene	20, 40, 100 and 200	SiHa	Cervix cancer	Collagenase treatment	Penetration after ECM degradation	[34]
				Size-switching		
Dendrimeric iCluster	100 → 5	BxPC-3	Pancreatic cancer	Size-switching, trigger = pH	Penetration Therapeutic activity	[43]
MSN WS2-HP Cluster Bomb	50 → 5	4T1	Breast cancer	Size-switching, trigger = pH	Penetration Therapeutic activity	[44]
Dendrimeric nanobomb	80 → 10	BxPC-3	Pancreatic cancer	Size-switching, trigger = pH	Penetration Therapeutic effect	[45]
PEG conjugated Multi-Micelles	80 → 4	BxPC-3	Pancreatic cancer	Size-switching, trigger = pH	Penetration Therapeutic effect	[46]
Gelatin	186.5 → 59.5	4T1 and B16F10	Breast cancer	Size-switching, trigger = matrix metalloproteinase-2	Penetration	[47]
Hyaluronic acid modified dendrimer	200 → 10	A549	Lung cancer	Size-switching, trigger = matrix metalloproteinase-2	Penetration	[48]
				Ligand functionalization		
PEG-PCL nanoparticle	120	C6	Brain cancer	iRGD functionalizationInterleukin-13 functionalization	Penetration	[49]
PLGA	112	4T1	Breast cancer	iRGD functionalization	Penetration Viability	[50]
HDL (lipoprotein) nanoparticle	136	A549	Lung cancer	iRGD functionalization	Penetration Viability	[51]
PLGA-*b*-PEG nanoparticle	107	C6	Brain cancer	CRT peptide functionalization, Tf receptor targeting	Penetration Viability	[52]
Elastin-like polypeptide nanoparticles	60	U-87	Brain cancer	Cell-penetrating peptide functionalization	Penetration	[53]
Folic acid-CM-PFA/pDNA	126–176	HeLa	Cervix cancer	Folic acid	Penetration pDNA expression Viability	[54]
			**Ligand functionalization (L) and size-Switching (SS)**			
Graphene quantum dot-loaded nanoparticle	150 → 5	RG2	Brain cancer	L: pH sensitive compound functionalization SS: trigger disassembly = irradiation with NIR light	Penetration	[55]
Lipid nanoparticle	180	BxPC-3	Pancreatic cancer	L: iRGD functionalization SS: trigger = hypoxia	Viability	[56]
				Nanoparticle shape		
Glycopolymer nanoparticle	sphere: 30 rod: 122 vesicle: 165	MCF-7	Breast cancer	Sphere/rod/vesicles	Penetration Viability	[57]
				Nanoparticle stiffness		
Fructose-based micelle nanorod	varies	MCF-7	Breast cancer	Stiff/soft	Penetration	[58]
polymer micelles	varies	BxPC-3	Pancreatic cancer	Stiff/soft	Penetration	[59]

^1^ Abbreviations used for cell lines: CALL-33: human tongue squamous carcinoma, HCT116: human colorectal carcinoma, KB: human epithelial carcinoma, MCF-7: human breast adenocarcinoma, 4T1: mouse breast carcinoma, 3T3: mouse fibroblasts, SiHa: human squamous cervix carcinoma, BxPC-3: human pancreatic adenocarcinoma, B16F10: mouse melanoma, T47D: human ductal carcinoma, C6: rat glioma, A549: human lung adenocarcinoma, U-87: human glioblastoma, RG2: rat glioma. Other abbreviations: PGLA: poly(glycolide-co-lactide), Au: gold, ECM: extracellular matrix, pDNA: plasmid DNA, MSN WS_2_-HP Cluster Bomb: mesoporous silica nanoparticle (MSN) capped with tumor-homing/penetrating peptide tLyP-1-modified tungsten disulfide quantum dots (WS_2_-HP), PEG: poly(ethylene glycol), PEG-PCL: poly(ethylene glycol)-poly(ε-caprolactone), HDL: high density lipoprotein, Folic acid-CM-PFA/pDNA: nanoparticles decorated with folic acid-poly(ethylene glycol) and dual amino acid-modified chitosan complexed with DNA.

**Table 2:** Overview of 3D model systems, their respective advantages and disadvantages and what knowledge they can provide in nanoparticle research.

Model	Advantages	Disadvantages	What Can we Learn?
Scaffold free spheroids	High-throughput, cheap Mimics solid tumors Only cell line and medium needed	No ECM interactions No vascularization	Nanoparticle penetration into solid tumors
Scaffold-embedded cells ^1^	Easy set-up of the experiment Mimics ECM-particle interactions	No vascularization Only a single cell Batch-to-batch variation (natural scaffold)	Nanoparticle-matrix interactions Nanoparticle diffusion Morphological changes at cellular level induced by the scaffold
Scaffold-embedded organoids ^1^	Cancer patient-specific Physiologically relevant Resembles in vivo tumor Tissue heterogeneity	No vascularization Requires skills, costly Batch-to-batch variation (natural scaffold)	Combination of nanoparticle-matrix interactions and nanoparticle-tumor tissue interactions Tumor heterogeneity Patient-specific testing
Micro-fluidics	Mimics vascularization “Building” the in vivo physiology using different cell types	Requires skills, costly Low throughput	Interactions with the stroma and the tumor tissue after the arrival of nanoparticles through the vasculature EPR effect

^1^ For all models embedded in a natural scaffold, the main disadvantage is associated with the batch-to-batch variation of the scaffold. For instance, Matrigel^®^ is produced by mouse sarcoma cells and the protein composition ratio varies between batches, changing the biochemical and mechanical properties of the material. In the case of synthetic scaffolds, the advantage is that one can control the scaffold stiffness parameter much better.
